# Association Between Childhood Behavioral Problems and Parental Military Active Duty Status: A Cross-Sectional Analysis of the 2017-2021 National Survey of Children’s Health

**DOI:** 10.7759/cureus.106299

**Published:** 2026-04-01

**Authors:** Kayla L Haydon, Vanessa C Kady, Chrisnel Lamy, Marcia Varella

**Affiliations:** 1 Pediatrics, Florida International University, Herbert Wertheim College of Medicine, Miami, USA; 2 Family Medicine, Florida International University, Herbert Wertheim College of Medicine, Miami, USA; 3 Epidemiology and Biostatistics, Florida International University, Herbert Wertheim College of Medicine, Miami, USA; 4 Medical and Population Health Sciences Research, Florida International University, Herbert Wertheim College of Medicine, Miami, USA

**Keywords:** behavioral health, conduct, mental health, military children, military-connected youth, military families

## Abstract

Introduction

Children of military families face unique stressors, including frequent relocations and parental deployments, which can disrupt stability and influence behavioral development. These experiences may also contribute to conduct problems and challenges in social interactions, including bullying victimization and perpetration. However, limited research exists specifically examining behavioral risks in military-connected children.

Objective

To assess whether children with at least one parent with current or prior military service have increased behavioral problems or a history of being bullied.

Methodology

We performed secondary data analysis of U.S. children aged 0-17 years who participated in the National Survey of Children’s Health (NSCH) from 2017 to 2021. The independent variable was parental military-duty status categorized as at least one parent with (1) current or (2) prior active-duty service in the U.S. Armed Forces, Reserves, or the National Guard, and (3) parents never in military duty (reference group). Outcomes assessed included (1) history of child behavioral or conduct problems and (2) history of being bullied. Outcomes were assessed independently. Confounding variables included demographic factors, family poverty ratio, deployment status, child’s general health, and a history of physical abuse and of living with mentally ill relatives. Logistic regression was used to estimate crude and adjusted associations.

Results

We analyzed data from 140,542 children: 4,919 (3.5%) had parents in active service, and 13,914 (9.9%) had parents with prior military service. A total of 10,681 (7.6%) reported a history of behavioral problems. Among those with information available regarding bullying (*n* = 81,382), 35,808 (44%) reported having ever been bullied. Children of currently active-duty parents were not associated with increased odds of behavioral problems (adjusted OR 0.89, 95% confidence interval (CI) 0.73-1.10, *P *= 0.289) but had higher odds of reporting being bullied (adjusted OR 1.30, 95% CI 1.07-1.58, *P *= 0.008), even after adjustment for selected covariates. Lastly, children of parents with prior military service had increased odds of both behavioral problems (adjusted OR 1.36, 95% CI 1.19-1.57) and being bullied (adjusted OR 1.19, 95% CI 1.08-1.31).

Conclusions

Parental military service was associated with increased risks of behavioral problems and risk of being bullied in U.S. children. Although we are unable to determine causation from this analysis, our findings support current literature highlighting the need for further research aimed at understanding the mechanisms and potential long-term health impacts of military service on child behavior. Behavioral health screening and early intervention programs could help to address emerging psychological and emotional challenges in this vulnerable population.

## Introduction

In 2016, military service was recognized as a cultural identity that can impact healthcare and health outcomes [1]. As children of parents serving in the military represent a growing population, it is important to consider the unique circumstances they experience while growing up in a military family.

According to the Department of Defense (DOD), the total number of military enlisted members and officers in 2022 was 2,071,451 [2]. Approximately 47.6% of members reported being married, and 37% reported having children [2]. This equates to 2,482,499 military family members in 2022, with 1,554,645 being children, of which the largest percentage (36.5%) were below five years of age [2].

Families with military service members have been reported to have increased risk of mental health conditions among adults and poorer academic achievement among their children [1]. In terms of child health, unique military-associated circumstances have been shown to impact child behavior and development [1]. First, military families and their children generally have mobility rates 2.4 times greater than civilian families, relocating to different geographical regions every few years [3]. Constant relocation results in a lack of continuity in healthcare, schooling, and supportive networks such as family ties and friend groups during a time when children are learning how to navigate these relationships [3]. Military children are more likely to struggle with educational and social transitions [4]. Relocation is also associated with increased psychiatric hospitalizations and poorer health outcomes, including high blood pressure and frequent headaches into adulthood [1]. As many military children start experiencing these challenges before the age of five, identification of mental and behavioral health concerns early on should be attempted and addressed.

Another unique challenge faced by military families is deployment. Children experience parental absence and separation for extended periods of time. These absences can trigger anxiety, which may manifest as tantrums, clinginess, or defiance [1]. Concern for a deployed parent's safety can further contribute to negative emotional outcomes [4]. Stress from both relocation and deployment has also been associated with increased rates of child maltreatment, including neglect and abuse [3]. These disruptions may occur during sensitive developmental periods, potentially altering the child's emotional regulation and coping mechanisms.

Mental health problems diagnosed early in childhood have detrimental effects on health and behavioral outcomes in young adulthood [5]. Notably, behavior often serves as the first observable sign of internal distress, particularly in young children who may not yet have the capacity to verbalize their emotions. Behaviors like aggression, withdrawal, or bullying can signal early emotional distress and may lead to chronic mental health issues if unrecognized [5]. This distinction is important because internalizing symptoms such as anxiety or depression may go undetected without clinical screening, whereas behavioral problems such as bullying are visible in everyday settings. Understanding how military-related stress manifests as externalized behaviors can inform targeted interventions and support culturally competent programs in both military and civilian school settings. Since many military children attend non-DOD schools where staff may be unfamiliar with their unique needs, recognizing these behaviors is essential to reducing isolation and fostering healthy peer relationships [3]. These insights can also guide broader efforts to expand behavioral health services and ensure systems of care evolve alongside the military community’s changing demographics.

While much literature discusses the association between military children and poor mental health outcomes, fewer studies address how these are manifested as behavioral problems, such as peer interactions and bullying. Thus, this study seeks to investigate if having at least one parent on military active duty was associated with increased behavioral problems, specifically bullying, in children aged 0-17 years old.

## Materials and methods

Design/Setting

We used cross-sectional data collected by the National Survey of Children’s Health (NSCH) from 2017 to 2021.

Sample

Participants consisted of children aged 0-17 years in the United States for whom information on key variables was available. Of the 175,231 children surveyed, 34,689 were excluded due to missing data for the exposure, outcome, or covariates required for each model. The final analytic sample consisted of 140,542 children.

Variables

All variables were based on parental self-reporting. Using NSCH caregiver items regarding whether either caregiver had ever served on active duty in the U.S. Armed Forces, Reserves, or National Guard, children were classified into three mutually exclusive groups: (1) current active-duty parental service, (2) prior parental active-duty service, or (3) no parental military service (reference).

Two outcomes were assessed independently. The first outcome was behavioral or conduct problems, defined using the NSCH question asking whether a doctor, other health care provider, or educator had ever told the parent or guardian that the child had behavioral or conduct problems. The second outcome was bullying victimization, defined using the NSCH question asking, "During the past 12 months, how often was this child bullied, picked on, or excluded by other children?" For analysis, both behavioral problems and bullying victimization were dichotomized as never versus any reported frequency. Because the bullying item is only asked for older children in the NSCH, this outcome was not assessed for children aged 0-5 years. We recognize that behavioral problems and bullying victimization are conceptually related but distinct constructs, and that the NSCH also includes a separate bullying-perpetration item not used as a primary outcome in this analysis.

Covariates were selected a priori based on prior literature and data availability in the NSCH. Adjusted models included child age group (0-5, 6-11, and 12-17 years), sex, race, and ethnicity category (as available in the dataset), household federal poverty level category, parental deployment history, child general health status, history of physical abuse, and history of living with a mentally ill household member.

Analysis

Exploratory and descriptive analyses were performed to assess participants’ characteristics and the overall frequency of behavior problems. Subsequently, bivariate analyses were conducted to compare distributions of each outcome across exposure groups and to assess the frequency of the outcomes according to those same characteristics. Lastly, binary logistic regression was used to estimate the odds of each outcome according to the exposure, before and after adjusting for confounders. Analyses were conducted using Stata v17 (StataCorp LLC, College Station, TX), applying survey procedures to account for the complex sampling design used by the original survey. Significance was considered for *P*-values ≤0.05. Effect modification by child’s age was tested by adding an interaction term to the model.

## Results

The total number of eligible study participants was 175,231. We excluded 34,689 participants due to missing values, resulting in a final sample size of 140,542. About 4,919 (3.5%) children had parents on active military duty, 13,914 (9.9%) had parents with prior military service, and 121,709 (86.6%) had parents without military service. Significant demographic differences were identified across these groups (Table 1). Children with currently active-duty parents were predominantly younger than those with formerly active-duty parents (39% vs. 27.6% aged 0-5 years). Children of households with current or prior active duty status were more likely to identify as Black or African American (15.2% current active duty vs. 15.4% prior active duty vs. 10.3% never) or multiracial (11.3% current active duty vs. 12.8% prior active duty vs. 8.7% never) (*P *< 0.001). Children of current active duty households were more likely to be male (54.1% male vs. 45.9% female, *P* < 0.019). Households with no history of active duty status were more likely (36.1%) to be >400% above the FPL as compared to current (29.2%) and prior (35.7) active duty status (P < 0.001). Of households with parents of current active duty status, 95.2% also reported a history of deployment. Comparatively, 75% of parents with prior active duty status reported a history of deployment (*P *< 0.001). There were differences in self-reported health status, with the highest percentage of children at 73.2% for current active duty status and the lowest at 66.1% of prior active duty status (*P* = 0.009). The highest percentage of children reporting exposure to physical abuse was 7.4% for those with prior active duty status parents, while the lowest percentage was 4.0% in those whose parents have never served in the military (*P* < 0.001).

**Table 1:** Baseline characteristics of children aged 0-17 years in the United States, based on parental active-duty status. AI/AN/A/NH/PI: American Indian, Alaska Native, Asian, Native Hawaiian, or Pacific Islander

Characteristics	Parental active duty status			*P*-value
	Current, *n* (%)	Prior, *n* (%)	Never, *n* (%)	
Age of the child				
0-5 years	1,921 (39.0)	3,513 (27.6)	42,972 (34.7)	<0.001
6-11 years	1,388 (31.5)	4,133 (32.3)	36,693 (33.7)	
12-17 years	1,667 (29.5)	6,483 (40.1)	44,206 (31.7)	
Race of the child				
White	3,869 (69.0)	10,946 (67.8)	99,579 (72.7)	<0.001
Black or African American	345 (15.2)	1,004 (15.4)	6,031 (10.3)	
AI/AN/A/NH/PI	250 (4.5)	570 (4.1)	8,741 (8.3)	
Two or more races	512 (11.3)	1,609 (12.8)	9,520 (8.7)	
Sex of the child				
Male	2,567 (54.1)	7,365 (50.8)	64,396 (51.2)	0.019
Female	2,409 (45.9)	6,764 (49.2)	59,475 (48.8)	
Household %FPL				
<100%	402 (11.1)	1,161 (11.2)	11,578 (15.0)	<0.001
100%-199%	821 (21.1)	2,103 (19.1)	17,867 (19.7)	
200%-399%	1,930 (38.6)	4,774 (34.0)	38,032 (39.2)	
400%+	1,823 (29.2)	6,091 (35.7)	56,394 (36.1)	
Deployment history of the parent				
Yes	1,299 (95.2)	2,312 (75.0)	NA	<0.001
No	44 (4.8)	732 (25.0)	NA	
Child’s general reported health				
Excellent	3,506 (73.2)	9,427 (66.1)	86,232 (68.2)	0.009
Very Good	1,107 (20.0	3,583 (24.8)	28,795 (23.6)	
Good	289 (5.5)	939 (8.0)	7,363 (7.1)	
Fair	56 (1.3)	127 (1.1)	1,078 (1.1)	
Poor	9 (0.1)	18 (0.0)	162 (0.1)	
Physical abuse experienced by the child				
Yes	201 (5.6)	860 (7.4)	4,362 (4.0)	<0.001
No	4,714 (94.4)	13,095 (92.6)	118,245 (96.0)	
Child living with a mentally ill relative				
Yes	415 (9.1)	1,756 (11.6)	9,071 (6.8)	<0.001
No	4,504 (90.9)	12,172 (88.4)	113,441 (93.2)	

Table 2 describes the prevalence of behavioral problems in children aged 0-17 years old based on selected demographic characteristics. Behavioral problems were reported in 10,826 (7.6%) children in our study. Children with parents with prior active duty status reported behavioral problems most frequently (10.2% prior active duty status vs. 6.41% current active duty status vs. 6.79% never). The frequency of behavioral problems was not significantly different based on the deployment history of the parent (*P *= 0.211). Frequency of behavior problems decreased with increasing socioeconomic status: 9.04% of children from households <100% of the FPL reported behavioral problems as compared to 5.76% of children from households >400% of the FPL (*P* < 0.001). Children with decreasing health status, history of physical abuse (25%), or history of living with a mentally ill family member (21.2%) were also more likely to present with behavioral problems than those who did not experience those factors (*P* < 0.001).

**Table 2:** Prevalence of behavioral problems in children aged 0-17 years, based on selected demographic characteristics. AI/AN/A/NH/PI: American Indian, Alaska Native, Asian, Native Hawaiian, or Pacific Islander

Characteristics	Behavioral problems		*P*-value
	No, *n* (%)	Yes, *n* (%)	
Active duty status of the parent			
Current	4,599 (93.6)	377 (6.4)	<0.001
Prior	12,610 (89.8)	1,519 (10.2)	
Never	114,941 (93.2)	8,930 (6.8)	
Age of the child			
0-5 years	46,980 (97.1)	1,426 (2.9)	<0.001
6-11 years	37,677 (90.1)	4,537 (9.9)	
12-17 years	47,493 (91.3)	4,863 (6.7)	
Race of the child			
White	105,805 (93.1)	8,589 (6.9)	<0.001
Black or African American	6,665 (90.9)	715 (9.1)	
AI/AN/A/NH/PI	9,103 (95.4)	458 (4.6)	
Two or more races	10,577 (91.4)	1,064 (8.6)	
Sex of the child			
Male	66,610 (90.2)	7,718 (9.8)	<0.001
Female	65,540 (95.8)	3,108 (4.2)	
Household %FPL			
<100%	11,736 (91.0)	1,405 (9.0)	<0.001
100%-199%	18,763 (91.8)	2,028 (8.2)	
200%-399%	41,323 (93.0)	3,413 (7.1)	
400%+	60,328 (94.2)	3,980 (5.8)	
Deployment history of the parent			0.211
Yes	3,260 (90.6)	351 (9.4)	
No	702 (93.3)	74 (6.7)	
Child’s general reported health			
Excellent	94,804 (96.0)	4,361 (4.0)	<0.001
Very Good	29,460 (88.7)	4,025 (11.3)	
Good	6,649 (79.9)	1,942 (20.1)	
Fair	867 (75.3)	404 (24.7)	
Poor	126 (66.7)	63 (33.3)	
Physical abuse experienced by the child			
Yes	3,917 (74.1)	1,506 (25.9)	<0.001
No	126,881 (93.8)	9,173 (6.2)	
Child living with a mentally ill relative			
Yes	8,742 (78.8)	2,500 (21.2)	<0.001
No	121,935 (94)	8,182 (6.0)	

Information regarding a history of being bullied was available for 81,382 participants, of whom 35,848 (44%) reported having ever been bullied (Table 3). Bullying was significantly higher in children with parents in active duty status (47.0% compared to 46.5% in those with formerly active duty and 40.3% in those whose parents never served, *P* < 0.001). There was no significant difference in the history of being bullied by the deployment status of the parent (*P* = 0.357). Children aged 6-11 years (47.3%) experienced higher bullying frequencies than adolescents aged 12-17 years (34.6%). Furthermore, males and females exhibited slightly different bullying rates (40.3% and 42.0%, respectively; *P* = 0.024). Black or African American children (32.5%) and American Indian, Alaska Native, Asian, Native Hawaiian, or Pacific Islander children (27.5%) reported lower bullying prevalence compared to White children (43.5%) (*P* < 0.001). Regarding socioeconomic status, children from households with increasing %FPL were more likely to report bullying (*P* < 0.001). Bullying occurrence increased with decreasing health status, history of physical abuse, and history of living with a family member with mental illness (*P* < 0.001).

**Table 3:** History of being bullied in children aged 0-17 years, based on selected demographic characteristics. AI/AN/A/NH/PI: American Indian, Alaska Native, Asian, Native Hawaiian, or Pacific Islander

Characteristics	History of being bullied		*P*-value
	No, *n* (%)	Yes, *n* (%)	
Active duty status of the parent			
Current	1,377 (53.0)	1,223 (47.0)	<0.001
Prior	4,792 (53.5)	4,283 (46.5)	
Never	39,365 (59.7)	30,342 (40.3)	
Age of the child			
0-5 years	NA	NA	<0.001
6-11 years	17,773 (52.7)	18,811 (47.3)	
12-17 years	27,761 (65.4)	17,037 (34.6)	
Race of the child			
White	35,146 (56.5)	30,184 (43.5)	<0.001
Black or African American	2,885 (67.5)	1,442 (32.5)	
AI/AN/A/NH/PI	4,108 (72.5)	1,512 (27.5)	
Two or more races	3395 (54.6)	2710 (45.4)	
Sex of the child			
Male	24,329 (59.7)	17,996 (40.3)	0.024
Female	21,205 (58.0)	17,852 (42.0)	
Household %FPL			
<100%	4,432 (65.4)	2,923 (34.6)	<0.001
100%-199%	6,713 (61.9)	5,098 (38.1)	
200%-399%	13,647 (56.6)	11,628 (43.4)	
400%+	20,742 (58.9)	16,199 (43.2)	
Deployment history of the parent			
Yes	1,330 (53.6)	1,172 (46.4)	0.357
No	210 (49.0)	193 (51.0)	
Child’s general reported health			
Excellent	32,959 (63.0)	21,035 (37.0)	<0.001
Very Good	9,725 (52.7)	10,721 (47.3)	
Good	2,421 (48.4)	3,433 (51.6)	
Fair	313 (43.1)	544 (56.9)	
Poor	49 (37.1)	72 (62.9)	
Physical abuse experienced by the child			
Yes	1,524 (41.5)	2,428 (58.5)	<0.001
No	43,461 (59.8)	33,091 (40.2)	
Child living with a mentally ill relative			
Yes	2,958 (38.4)	4,943 (61.6)	<0.001
No	41,979 (60.8)	30,556 (39.2)	

Children of parents with prior military service had significantly higher odds of behavioral problems (unadjusted OR 1.57, 95% CI 1.38- 1.77, *P* < 0.001), whereas having a parent of current active duty was not significantly associated with behavioral issues (unadjusted OR 0.94, 95% CI 0.78-1.14, P = 0.526) (Table 4). After adjustment for household % FPL, sex, race, age, health status, and exposures to physical abuse and mental illness, children of parents with prior active duty status were still associated with increased odds of behavioral problems (adjusted OR 1.36, 95% CI 1.19-1.57, P < 0.001). Children with parents of current active duty status remained not significantly associated with behavioral problems post-adjustment (adjusted OR 0.89, 95% CI 0.73-1.10, P = 0.289). The deployment history of the parent was not significantly associated with increased behavioral issues in unadjusted models. Males had higher odds of behavioral problems (adjusted OR 2.63, 95% CI 2.38-2.91, P < 0.001), and older children, especially those aged 6-11 (adjusted OR 3.41, 95% CI 1.77-6.55, P < 0.001), had the highest odds for this outcome. American Indian, Alaska Native, Asian, Native Hawaiian, or Pacific Islander children had lower odds of behavior problems compared to White children (adjusted OR 0.61, 95% CI 0.47-0.78, P = 0.009). Poor health significantly increased the likelihood of behavioral problems (adjusted OR 1.80, 95% CI 1.02-3.17, p = 0.042), whereas excellent or very good health was protective. Additionally, a history of physical abuse (adjusted OR 1.97, 95% CI 1.12-3.46, P < 0.001) and living with a mentally ill family member (adjusted OR 2.62, 95% CI 2.30-3.00, P < 0.001) were independently associated with increased odds of behavioral problems.

**Table 4:** Unadjusted and Adjusted associations between behavioral problems, active duty status of parent, sex of child, race of child, deployment history of parent. AI/AN/A/NH/PI: American Indian, Alaska Native, Asian, Native Hawaiian, or Pacific Islander

Characteristics	Unadjusted		Adjusted	
	OR (95% CI)	p-value	OR (95% CI)	p-value
Active Duty Status of Parent				
Current	0.94 (0.78,1.14)	0.526	0.89 (0.73,1.10)	0.289
Prior	1.57 (1.38,1.77)	<0.001	1.36 (1.19,1.57)	<0.001
Never	Ref		Ref	
Age of Child				
0-5 years	Ref		Ref	
6-11 years	3.74 (3.25,4.29)	<0.001	3.41 (1.77,6.55)	<0.001
12-17 years	3.24 (2.82,3.72)	<0.001	2.50 (1.29, 4.86)	0.007
Race of Child				
White	Ref		Ref	
Black or African American	1.35 (1.17,1.56)	<0.001	1.09 (0.93,1.82)	0.272
AI/AN/A/NH/PI	0.65 (0.50-0.83)	0.001	0.61 (0.47,0.78)	0.009
Two or more races	1.27 (1.10,1.48)	0.002	1.14 (0.96,1.34)	0.127
Sex of Child				
Male	2.47 (2.25,2.72)	<0.001	2.63 (2.38,2.91)	<0.001
Female	Ref		Ref	
Household %FPL				
<100%	1.62 (1.41,1.87)	<0.001	1.02 (0.87,1.20)	0.782
100-199%	1.45 (1.29,1.63)	<0.001	1.01 (0.89, 1.14)	0.933
200-399%	1.24 (1.12,1.37)	<0.001	0.98 (0.89,1.09)	0.774
400%+	Ref		Ref	
Deployment History of Parent				
Yes	1.45 (0.81, 2.62)	0.213	1.75 (0.97,3.17)	0.062
No	Ref		Ref	
Child’s General Reported Health				
Excellent	0.16 (0.14,0.19)	<0.001	0.19 (0.16,0.22)	<0.001
Very Good	0.50 (0.44,0.58)	<0.001	0.52 (0.44,0.61)	<0.001
Good	Ref		Ref	
Fair	1.30 (0.98,1.72)	0.071	1.29 (0.95,1.74)	0.098
Poor	1.98 (1.08,3.63)	0.027	1.80 (1.02,3.17)	0.042
Child Experienced Physical Abuse				
Yes	5.30 (4.59,6.11)	<0.001	1.97 (1.12, 3.46)	<0.001
No	Ref		Ref	
Child Lived with Mentally Ill Relative				
Yes	4.25 (3.81,4.73)	<0.001	2.62 (2.30, 3.00)	<0.001
No	Ref		Ref	

When assessing the outcome of having a history of being bullied (Table 5), children of current and prior active duty parents exhibited higher odds of being bullied compared to those with no military-affiliated parents, pre-adjustment for confounders (unadjusted OR 1.32, 95% CI 1.10-1.56, P = 0.003 and unadjusted OR 1.29, 95% CI 1.17-1.42, *P* < 0.001, respectively) and post-adjustment (adjusted OR 1.30, 95% CI 1.07-1.58, *P* = 0.008 and adjusted OR 1.19, 95% CI 1.08-1.31, *P* < 0.001, respectively). Parental deployment history was not significantly associated with a history of being bullied. Age was strongly associated with the odds of being bullied. Children aged 6-11 have over twice the odds of being bullied compared to younger children (adjusted OR 2.18, 95% CI 1.61-2.94, *P* < 0.001). Male children had slightly lower odds of being bullied than females (adjusted OR 0.92, 95% CI 0.87-0.98, *P* = 0.014). Regarding race, Black or African American children (adjusted OR 0.64, 95% CI 0.57-0.72, *P* < 0.001) and children of American Indian, Alaska Native, Asian, Native Hawaiian, or Pacific Islander descent (adjusted OR 0.51, 95% CI 0.43-0.56, *P* < 0.001) had significantly lower odds of being bullied compared to White children. Contrary to the association with behavioral problems, lower household socioeconomic status was associated with a decreased history of being bullied. Children from lower-income households (<100% FPL) had significantly lower odds of behavioral problems compared to those from higher-income households (400%+ FPL) (adjusted OR 0.60, 95% CI 0.54-0.68, *P* < 0.001). General health status also played a role, as children reported to be in excellent (adjusted OR 0.51, 95% CI 0.45-0.59, *P* < 0.001) or very good health (adjusted OR 0.82, 95% CI 0.72-0.95, *P* = 0.007) had lower odds of being bullied compared to those in good health. Notably, children who had experienced physical abuse (adjusted OR 1.63, 95% CI 1.39-1.92, *P* < 0.001) or lived with a mentally ill family member (adjusted OR 2.01, 95% CI 1.78-2.26, *P* < 0.001) had significantly higher odds of being bullied.

**Table 5:** Unadjusted and adjusted associations between history of being bullied and parental active-duty status, child sex, child race, and parental deployment history. AI/AN/A/NH/PI: American Indian, Alaska Native, Asian, Native Hawaiian, or Pacific Islander

Characteristics	Unadjusted		Adjusted	
	OR (95% CI)	*P*-value	OR (95% CI)	*P*-value
Active duty status of the parent				
Current	1.32 (1.10-1.56)	0.003	1.30 (1.07-1.58)	0.008
Prior	1.29 (1.17-1.42)	<0.001	1.19 (1.08-1.31)	<0.001
Never	Ref		Ref	
Age of the child				
0-5 years	Ref		Ref	
6-11 years	1.70 (1.59-1.80)	<0.001	2.18 (1.61-2.94)	<0.001
12-17 years	Unavailable		Unavailable	
Race of the child				
White	Ref		Ref	
Black or African American	0.63 (0.56-0.70)	<0.001	0.64 (0.57-0.72)	<0.001
AI/AN/A/NH/PI	0.49 (0.42-0.58)	0.001	0.51 (0.43-0.56)	<0.001
Two or more races	1.08 (0.95-1.23)	0.256	1.00 (0.87-1.15)	0.989
Sex of the child				
Male	0.93 (0.88-0.99)	0.024	0.92 (0.87-0.98)	0.014
Female	Ref		Ref	
Household %FPL				
<100%	0.69 (0.62-0.77)	<0.001	0.60 (0.54-0.68)	<0.001
100%-199%	0.81 (0.74-0.88)	<0.001	0.73 (0.66-0.80)	<0.001
200%-399%	1.01 (0.94-1.08)	0.781	0.93 (0.86-1.00)	0.047
400%+	Ref		Ref	
Deployment history of the parent				
Yes	0.83 (0.56-1.23)	0.358	0.73 (0.47-1.12)	0.148
No	Ref		Ref	
Child’s general reported health				
Excellent	0.55 (0.49-9.63)	<0.001	0.51 (0.45-0.59)	<0.001
Very Good	0.84 (0.73-0.97)	0.015	0.82 (0.72-0.95)	0.007
Good	Ref		Ref	
Fair	1.24 (0.85-1.81)	0.255	1.25 (0.82-1.89)	0.293
Poor	1.59 (0.82,3.08)	0.166	1.63 (0.84, 3.16)	0.146
Physical abuse experienced by the child				
Yes	2.09 (1.79-2.44)	<0.001	1.63 (1.39-1.92)	<0.001
No	Ref		Ref	
Child living with a mentally ill relative				
Yes	2.49 (2.23-2.78)	<0.001	2.01 (1.78-2.26)	<0.001
No	Ref		Ref	

## Discussion

The objective of this study was to assess whether children aged 0-17 years, with at least one parent with current or prior military service, have increased behavioral problems, especially related to bullying, as compared to children with parents without a history of military service. Our results indicate that a history of prior military service of a parent was associated with increased odds of both behavioral problems and being bullied. Children of parents with current active duty service were not associated with a higher risk of behavioral problems, but they were found to have a higher risk of being bullied.

These results of increased odds of behavior problems in children with parents with prior military service are consistent with other studies. For instance, a cross-sectional analysis of 2018-2019 data from the National Survey of Children’s Health (NSCH) comparing 4,028 school-aged children from veteran families with 38,228 children from non-veteran families showed that children of veteran families have increased rates of externalizing behavioral conditions (RR 1.42, 95% CI 1.21-1.66, *P *< 0.001) [6]. Yet, we found that children with parents in current active duty status were not associated with behavioral problems. Such findings contrast with those of Williamson et al.’s 2016-2018 systematic review, which found that military-connected youth were more likely to get into physical fights (OR 1.67, 95% CI 1.62-1.71) or carry a weapon (OR 1.90, 95% CI 1.83-1.97) [7]. The differences in our results could potentially be attributed to how behavioral problems are defined in the NSCH. The NSCH survey does not specify fighting or carrying weapons. Additionally, the systematic review could potentially have had higher statistical power to detect that association. Of note, while current active duty status was not associated with increased risk of behavioral problems, it was associated with increased risk of being bullied. This is in accordance with the results of a 2016-2018 qualitative analysis of 24 students in grades 7-12 from Southern California, which found that children with parents on current active duty were more likely to report a history of being bullied [8].

Regarding the impact of parental deployment status on behavioral problems, we found no significant associations between parental deployment status and history of behavioral problems or bullying. In 2016, Mustillo et al. published results from a cross-sectional study of 680 children for whom increased peer and emotional problems were reported in children aged 6 to 10 years with parents who were deployed during the child’s birth [9]. Similarly, in the systematic review by Williamson et al. mentioned above, it was also found that children with deployed parents were more likely to experience adjustment difficulties and display externalizing behaviors [7]. Differences in study methodology might explain this inconsistency. For example, Mustillo et al.’s study data collection included a telephone interview, which may be easier to elicit answers regarding more sensitive topics such as behavioral problems [9]. Williamson et al.’s 2016-2018 systematic review included studies utilizing self-reported measures administered directly to children in school settings [7], whereas the NSCH is administered to parents in the household setting. Whether the NSCH was less sensitive for detecting behavioral problems, and/or it had less statistical power, is yet to be confirmed.

Other incidental findings of this study were that behavioral problems were more prevalent in males and older children, particularly in adolescents, underscoring the cumulative impact of prolonged exposure to stress and family instability. However, these older children (12-17 years) exhibited reduced odds of bullying relative to their younger counterparts (6-11 years). This aligns with developmental trajectories indicating a peak in bullying incidents during middle school, followed by a decline as adolescents refine coping mechanisms and social strategies [10]. Male sex was found to be associated with higher odds of behavioral problems, aligning with established findings that boys are more prone to externalizing behaviors, including aggression and hyperactivity, in response to chronic stress and environmental instability [11]. 

Several other covariates were strongly associated with both outcomes, including poorer child health, physical-abuse exposure, and living with a mentally ill household member. Poorer self-reported health and histories of exposure to physical abuse and mental illness markedly increased the likelihood of both outcomes, consistent with robust evidence linking adverse childhood experiences to heightened psychosocial risk profiles [6]. Parental mental health issues are well-documented drivers of family dysfunction, which may indirectly amplify children's exposure to peer victimization and behavioral maladaptation [12]. Our findings reinforce that childhood behavioral and social difficulties are multifactorial. Although we adjusted for several important sociodemographic and family-level factors, residual confounding is likely. Of note, household socioeconomic status was not found to be associated with increased odds of behavioral problems post-adjustment, which is inconsistent with Williamson et al.'s study that reported financial strain to be associated with exacerbation of childhood adversities, including bullying exposure and behavioral disturbances [7].

The results of this study were based on secondary analyses of cross-sectional data collected from the child’s parents, thus precluding causal inference. The outcomes from the NSCH are parent-reported, which may lead to recall bias, underreporting, and differential misclassification, particularly for instances of bullying or behavioral concerns that children may not disclose to caregivers. Prior studies have described that parents report on the history of their child’s defiant behaviors and emotional problems are underestimated [13]. Furthermore, male and minority children were also found to underreport bullying [14]. Another limitation relates to selection bias. Military-affiliated families frequently relocate within the United States or may be located outside of the United States, potentially resulting in lower response rates and under-representation of those deployed and, potentially, at higher risks [15]. Furthermore, children with parents who passed away during combat would be likely less represented in this survey, limiting its external validity.

Several potential factors were not available in the NSCH to be accounted for. We analyzed the history of bullying victimization as a dichotomized outcome, so we are unable to ascertain frequency, severity, and timing. The reporting of prior military service also does not capture heterogeneity of duration, location, and responsibilities during service. Mustillo et al. reported that timing, duration, and location of deployment impacted the prevalence of peer problems and emotional problems [8]. Rather than establishing directionality, the present findings identify patterns of association that may help guide screening awareness and future longitudinal research. Studies with more robust data on child-reported outcomes, familial environment, school environment, transition after military separation, and post-service stressors are needed to clarify mechanisms and identify modifiable targets for intervention.

## Conclusions

In conclusion, our study found that military service is associated with increased behavioral problems and bullying in children in the United States. Although we are unable to determine causation from this analysis, our findings support current literature highlighting the need for further research aimed at understanding the mechanisms and potential long-term health impacts of military service on child behavior. Future studies should also aim to evaluate interventions to successfully minimize the risk of behavioral problems. Further knowledge in the field can guide the continued development of multidisciplinary, evidence-based interventions tailored to all military-connected children. Policies aimed at enhancing mental health resources, bolstering school-based support services, and ensuring continuity of care during military relocations are critical. Additionally, behavioral health screening and early intervention programs should be prioritized to address emerging psychological and emotional challenges in this vulnerable population.
